# Age and vascular aging: an unexplored frontier

**DOI:** 10.3389/fcvm.2023.1278795

**Published:** 2023-11-09

**Authors:** Laura del Mar González, Sara P. Romero-Orjuela, Fernando J. Rabeya, Valeria del Castillo, Darío Echeverri

**Affiliations:** ^1^ Department of Cardiology, Fundación Cardioinfantil–Instituto de Cardiología, Bogotá, Colombia; ^2^ School of Medicine and Health Sciences, Universidad del Rosario, Bogotá, Colombia

**Keywords:** vascular age, vascular stiffness, aging, arterial stiffness, risk factors, cardiovascular diseases, pulse wave analysis

## Abstract

Vascular age is an emerging field in cardiovascular risk assessment. This concept includes multifactorial changes in the arterial wall, with arterial stiffness as its most relevant manifestation, leading to increased arterial pressure and pulsatile flow in the organs. Today, the approved test for measuring vascular age is pulse wave velocity, which has been proven to predict cardiovascular events. Furthermore, vascular phenotypes, such as early vascular aging and “SUPERNOVA,” representing phenotypic extremes of vascular aging, have been found. The identification of these phenotypes opens a new field of study in cardiovascular physiology. Lifestyle interventions and pharmacological therapy have positively affected vascular health, reducing arterial stiffness. This review aims to define the concepts related to vascular age, pathophysiology, measurement methods, clinical signs and symptoms, and treatment.

## Introduction

Aging is a complex and irreversible biological process affecting living beings. Highly predictable molecular and cellular damage accumulates over time, causing progressive deterioration of physical and mental abilities and increasing susceptibility to diseases and the risk of death (1). Aging has proven to be a heterogeneous process since the organs of an individual have different aging processes that may be affected by genetic, environmental, and/or lifestyle habit factors (2).

In 2008, the concept of vascular age (VA), also known as “heart age,” was described in the Framingham study. In this study, D'Agostino et al. (3) defined it as the absolute cardiovascular risk (CVR) according to the age of a person if all modifiable risk factors (RFs) were controlled. This risk inherent to age is a consequence of vascular aging (VAg), which causes structural and functional changes in the vessels (4). This definition was established to help patients understand their CVR, especially young people with intermediate CVR and poor adherence to preventive measures (5). Recently, studies have shown a correlation between VA and various diseases such as dementia, chronic kidney disease (CKD), and metabolic syndrome. Moreover, VA has been identified as a predictive factor for increased CVR (6, 7).

At the cardiovascular (CV) level, aging is characterized by endothelial dysfunction, vascular remodeling, and loss of arterial distensibility (8, 9), showing increased vessel wall stiffness and elevated systolic arterial pressure (SAP) and pulse pressure (PP) (10). These changes in the arterial wall contribute to what is currently known as VAg, described as a natural and progressive biological aging phenomenon that involves biochemical, cellular, and enzymatic events. Its progression determines organ function throughout life (11).

## Materials and methods

An article search was conducted on PubMed and Google Scholar, using the keywords in plain and MeSH terms. The search was limited to articles published in English and those published between 2015 and 2022. After removing duplicates, the articles were selected by title and abstract. Those selected were downloaded as full-text documents for careful reading, resulting in 19 articles with the essential characteristics for inclusion in the review. The bibliography of each selected article was reviewed to find additional articles that would contribute to the review. The articles which were ultimately included were incorporated into a narrative.

## Normal arterial wall

During the embryonic period, in the third week of development, the blood vessels are formed from the mesoderm through vasculogenesis (blood vessels arising from blood islands, which are derived from mesodermal cells) and angiogenesis (sprouting from existing vessels) (12). The first blood islands form approximately in the third week of gestation, surrounding the yolk sac and appearing in the lateral plate mesoderm and other areas. The CV system appears toward the fourth week of development.

The arterial wall comprises three layers, namely, tunica intima, tunica media, and adventitia. The tunica intima is composed of the endothelium, a basement membrane, and an internal elastic lamina with fenestrations that allow substances to pass between the various layers (13).

The tunica media is the thickest layer and is the most variable in the different types of vessels. It comprises lamellar units containing elastic lamellae and an interlamellar space with smooth muscle, elastin fibers, collagen (types I and III), and proteoglycans (8, 14). This tunica has vasoconstriction and vasodilation capabilities, which are important characteristics for blood flow regulation (15). Pericytes are mural cells surrounding the endothelium in blood vessels, especially arterioles, venules, and capillaries. The pericyte-to-endothelial cell ratio varies from tissue to tissue, with a higher concentration in the blood‒brain barrier (16).

Pericytes perform various functions such as angiogenesis, greater blood vessel support, flow regulation, maturation, vascular remodeling, and vessel patency. Pericytes have been reported to secrete large amounts of substances such as immune regulating factors, angiogenic growth factors, quiescence-inducing factors, and extracellular matrix (ECM) (17, 18).

One of the most important functions of pericytes is the expression of angiogenic factors such as transforming growth factor beta (TGF-β), angiopoietin-1, and vascular endothelial growth factor, leading to the differentiation and proliferation of both pericytes themselves and endothelial cells (ECs). In addition to promoting the formation of new vessels, these cells also help regenerate and repair tissues by generating ECM and associated factors (17).

Pericytes can act as parent cells, differentiate into vascular smooth muscle cells (VSMCs), and express alpha-actin and myosin, which gives them properties similar to those of other mural cells. These functions regulate microcirculatory blood flow through contractility and dilation (16, 18).

The adventitia is the outer layer and is made up of loose connective tissues essential for connecting the arteries to other body tissues, including nerves, lymph vessels, and smaller blood vessels, keeping the arteries in their place and maintaining adequate CV functioning.

There are three types of arteries, namely, elastic, muscular, and arterioles. Elastic or conducting arteries are the largest, with a thicker layer of elastic tissues in their tunica media. This layer helps buffer the pulsatile flow caused by heart contraction, turning it into a more regular flow and allowing the Windkessel effect (13, 15).^[1]^ Muscular or distribution arteries have predominantly muscular layers in their tunica media and a greater capacity for vasoconstriction or vasodilation, adjusting blood flow velocity as blood is distributed to the organs. Finally, the arterioles consist of smooth muscle and lack a significant elastic lamina. The arteriolar walls increase friction between the vessel wall and the blood and cause greater resistance to coronary flow (13).

## Pathophysiology of vascular aging

Arterial stiffness (AS) accompanies humans throughout their life cycle. Studies have shown that the first wall changes appear during uterine (20) and early postnatal life, influenced by RFs such as fetal growth and prematurity (21). An inverse association has also been found between birth weight adjusted for gestational age and elevated SAP in childhood, adolescence, and adulthood, along with higher CVR (6).

Vascular aging describes a process mediated by structural and functional modifications in the vessel wall. It is characterized by endothelial dysfunction leading to increased vascular smooth muscle tone, favoring the release of vasoactive substances, which increase its tone, decrease arterial distensibility, and deteriorate the arteries' capacity to adapt to blood pressure (BP) changes (22). Increased AS has been described as one of the first detectable wall abnormalities and is also considered the hallmark of VAg (23).

**Figure 1:**
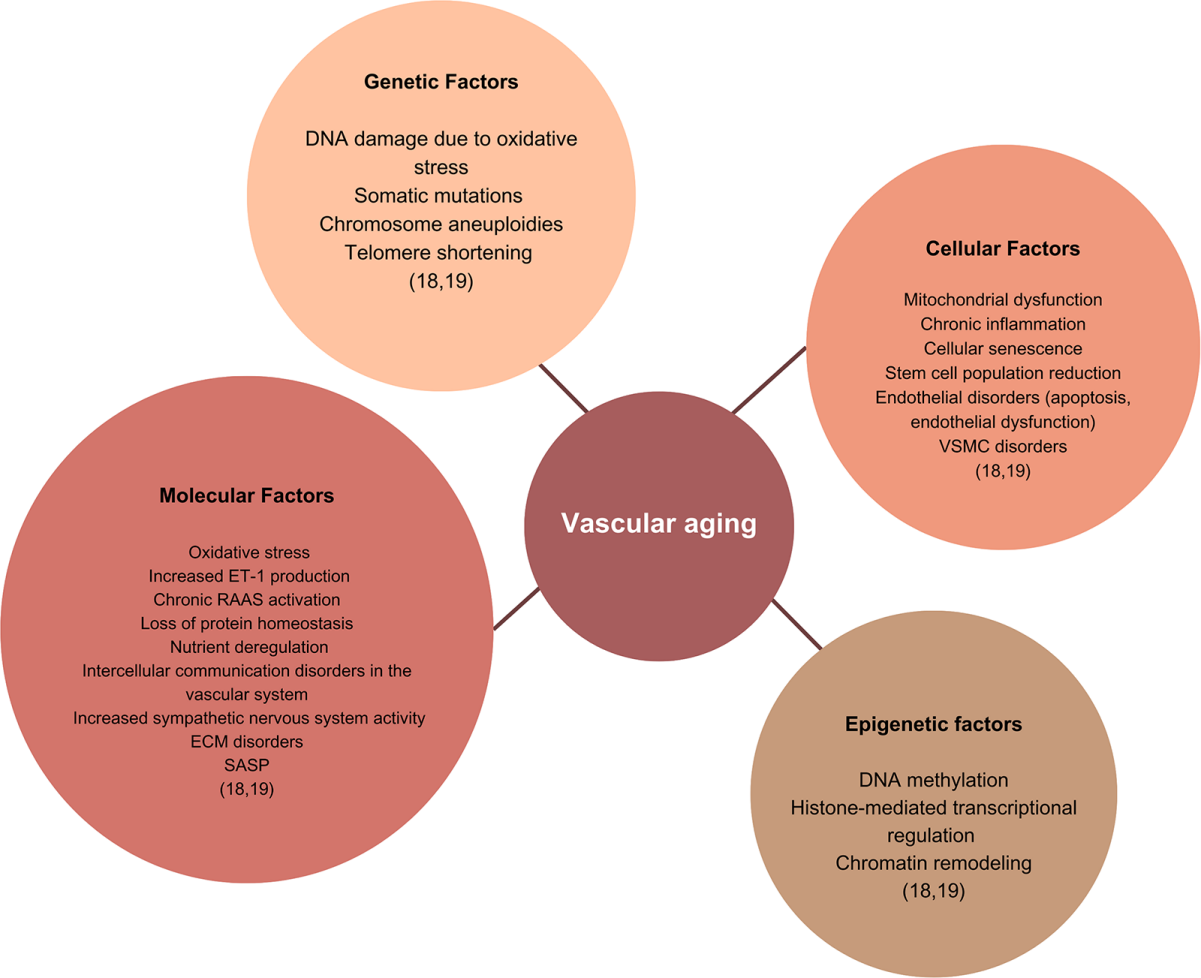
Factors involved in the pathophysiology of VAg. The figure shows the factors involved in the pathogenesis of vascular aging, grouped by etiology as molecular, epigenetic, genetic, and cellular. DNA, deoxyribonucleic acid; ET-1, endothelin 1; RAAS, renin–angiotensin–aldosterone system; ECM, extracellular matrix; VSMC, vascular smooth muscle cell; SASP, senescence-associated secretory phenotype.

Many factors are involved in the pathophysiology of VAg (Figure 1), which affect the course of AS, especially oxidative stress, chronic low-grade inflammation, and structural changes in the vascular smooth muscle endothelium and ECM (22). There are also other factors such as the biomechanics recognized as mechanical homeostasis between ECM and VSMCs, which is a fundamental concept in AS (24), the plasticity of the VSMCs, vascular tone, VSMC stiffness, and activity of the renin–angiotensin–aldosterone system.

### Vascular aging and oxidative stress

The vascular endothelium is essential for tone and regulation of blood flow by releasing vasodilating molecules such as nitric oxide (NO) (8, 25). The ECs are the main source of NO production through endothelial NO synthase activation, induced by mechanical and chemical stimulation and generated by metabolic conversion of L-arginine to L-citrulline (8, 25, 26). NO regulates blood vessel distensibility, exhibits an anti-inflammatory effect, and can even function as an intracellular messenger in the vessel wall (20, 25).

Oxidative stress and proinflammatory signaling are important factors that promote AS, causing increased vascular smooth muscle tone by reducing the bioavailability of NO (8, 10, 27). The decreased bioavailability of NO secondary to endothelial dysfunction affects the vasodilating properties of the endothelial barrier (10), contributing to reduced elasticity and increased AS, especially in conducting arteries, causing BP elevation. When there is an excess of reactive oxygen species (ROS), especially superoxide (O^2−^), among the ECs, these molecules react directly with NO, causing deactivation and the formation of more ROS (28).

### Vascular aging and inflammation

Inflammation is an important RF for AS and VAg progression (9, 17, 29). Epidemiological studies have shown that aging-related inflammation is an RF for CV disease (CVD), CKD, cancer, dementia, and depression as well as an indicator of frailty and premature death (30). Inflammatory markers and biomarkers with proatherogenic properties such as C-reactive protein (CRP), adiponectin, nuclear factor-κB, and insulin-like growth factor 1 are involved in the pathogenesis of vessel damage, contributing to worse CV outcomes (31).

Elevated CRP and decreased adiponectin levels are independent RFs for developing AS. In addition, people with high CVR tend to have elevated high-sensitivity CRP levels compared with patients with low CVR (9, 31). With age, deregulating these central inflammatory mediators and factors such as nuclear factor-κB in the ECs contributes to endothelial function abnormalities, favoring oxidative stress and VAg (25, 31).

People with chronic inflammatory diseases such as rheumatoid arthritis, inflammatory bowel disease, and human immunodeficiency virus (HIV) have been found to have more AS than the healthy population (29, 32). AS is characterized by significant chronic inflammation involved in cardiac and vascular remodeling, representing an important RF for fatal and non-fatal CV events (29). Patients with AS also have a greater predisposition to acute myocardial infarction (AMI) than the healthy population, showing the same tendency as people 10 years older (32).

### Structural changes of vascular aging

Over time, the endothelial barrier loses its integrity, and inflammation, hypoxia, and mechanical factors, among others, lead local totipotent cells to differentiate into smooth muscle cells (VSMCs) that increase their proliferation, migration, apoptosis, and senescence capacity, progressively invading the subendothelial space. Once there, these cells continue synthesizing ECM components, which results in intimal thickening. In addition, with age and the related epigenetic changes, VSMCs lose their contractility, a phenomenon which leads the forces exerted upon the wall to be poorly distributed between collagen and elastin molecules, leading to the AS found with aging which, in addition, is an independent RF for atherosclerosis and future CVD-related events (10, 33).

**Figure 2:**
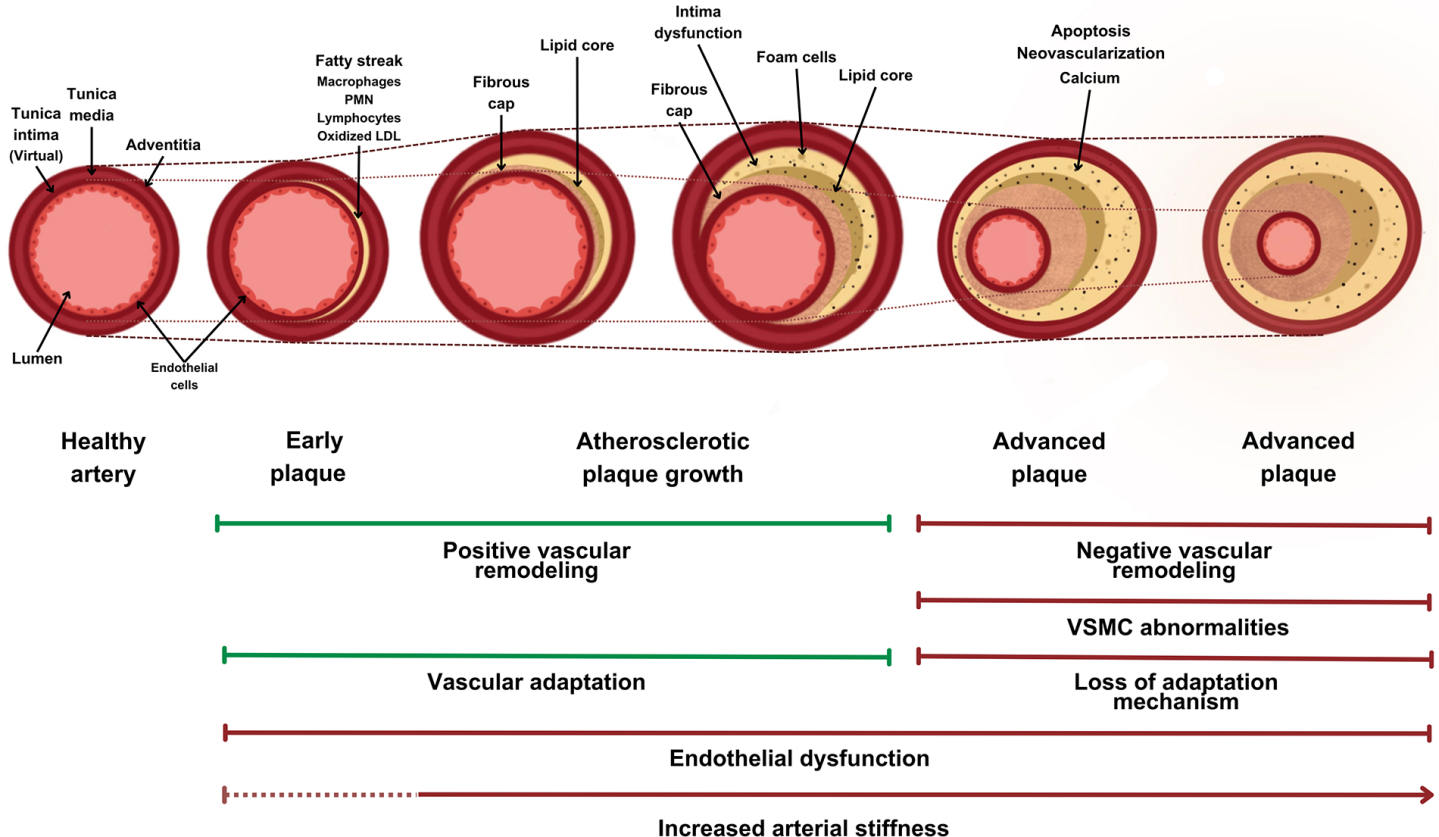
Structural changes in atherosclerotic arteries and their relationship with vascular stiffness. Structural changes in arteries undergoing an arteriosclerotic process, showing their ability to adapt and the structural plaque changes. Over time, there is negative vascular remodeling, affecting the lumen. The dotted lines show changes in the vessel area over time and changes in the luminal area due to vascular remodeling. PMN, polymorphonuclear neutrophils; LDL, low-density lipoprotein; VSMC, vascular smooth muscle cell.

The ECM components give the vessel wall its adaptability and intrinsic resistance. The ECM is remodeled throughout life, with help from the VSMCs, which participate in its synthesis, repair, and maintenance. With aging, the number of VSMCs is reduced, contributing to the loss of ECM structural and functional integrity (10). Remodeling also occurs, increasing collagen deposits, elastin degradation (1), and formation of advanced glycation end products, leading to increased AS (34) through structural protein crisscrossing (Figure 2) (25, 31, 32).

Pericytes play a key role in blood vessel fibrosis, calcification, and inflammation and are more concentrated in atherosclerotic sites. Pericytes contribute to the structural formation of plaque and neovascularization in the vessel walls. Under the stimulation of proinflammatory cytokines, pericytes could lead to neutrophil-mediated immune activation, promoting atherosclerosis. However, their specific role in the progression of VA is yet to be known. Further studies are needed to assess their role in this process (17, 18).

### Epigenetic factors

Three main types of epigenetic regulation affect VA. The first is DNA methylation, which involves binding the methyl or hydroxymethyl groups to the GpC islands, producing 5-methylcytosine molecules through cytosine methylation, which regulates gene expression in promoter regions. Thus, a large number of these molecules (hypermethylation) downregulate gene expression, and their absence (hypomethylation) promotes gene expression (35).

This regulation occurs through different enzymes known as DNA methyltransferases or serine hydroxymethyltransferases when they bind hydroxymethyl groups. The expression of some of these has been related to aging and the onset of CVD. For example, in mouse models, a relationship has been found between dysfunction in Dnmt3 and TET-2 (a serine hydroxymethyltransferase) enzymes in hematopoietic cells and inflammatory deregulation of myeloid cells, leading to heart muscle hypertrophy, arterial fibrosis, and diminished cardiac function (35, 36).

Second, histone structure modifications play an important epigenetic role by altering the three-dimensional structure of chromatin and regulating gene expression. Many enzymes regulate this process, several of which have been related to vascular inflammation, endothelial dysfunction, and vascular aging. Histone deacetylase 4 has been shown to play a key role in regulating vascular inflammatory pathways and avoiding endothelial dysfunction by regulating endothelial cell autophagy (35, 37, 38).

Third, non-coding RNAs are RNA molecules classified according to the number of nucleotides they contain, categorized into short ncRNA, long ncRNA, circular RNA, and microRNA. They regulate cellular translation through interactions with messenger RNA, such as the case of microRNAs, which inhibit cellular translation by binding to the nucleotide chain, keeping it from entering the ribosome and thus limiting protein expression. This has been related to CV health, with miR-22 and miR-12-3p related to VSMC proliferation, contractility, differentiation, and migration (39, 40).

## Measuring vascular age

There are two main methods for estimating VA. The first method is based on AS percentiles according to age and sex obtained from healthy people (6), calculated using techniques such as carotid–femoral pulse wave velocity (cf-PWV). The second method is based on the CVR determined by risk scales such as the Systematic Coronary Risk Evaluation (SCORE), Framingham Score, ASSING, Reynolds, QRISK, PROCAM, HEARTS, ACC/AHA, and ASCVD.

Pulse wave velocity, or cf-PWV, is the speed at which the pressure wave travels through the aorta and great arteries during each cardiac cycle (18). The measurement of cf-PWV is considered the gold standard for assessing AS and has important epidemiological evidence of its predictive value for CV events (17, 31). AS is measured through aortic stiffness, measured using cf-PWV, whose normal values are generally considered to be <10 m/s (34, 41). When determining the normal range, population differences and age must also be considered since it can vary from 6.2 m/s to 10.9 m/s, depending on the age of the patients (42). It is calculated using the following equation:

$$\mathrm{PWV}\;=\frac{D(m)}{t(s)}$$

where “*D*” is the distance between the recording sites and “*t*” is the time taken by the arterial pulse to travel this distance (18).

The pulse wave is formed after a heart contraction and travels along the arterial bed until it meets peripheral resistance (usually at a bifurcation), which will determine the appearance of the wave (31). In young people, the reflected wave is slow and occurs during diastole due to greater arterial elasticity (1, 31, 34). This translates into increased diastolic arterial pressure (DAP) and, as a result, indicates adequate coronary perfusion (34). Finally, the wave reflection returns part of the pulsatile energy toward the central aorta, where it dissipates and limits transmission of this energy to the periphery, avoiding microcirculation damage. With VAg, cf-PWV increases, seen as a change in the pulse wave, which results in an early reflection (1). As a result, the wave reaches the heart during systole, increasing SAP and cardiac output, and may also affect coronary perfusion (1, 31).

Other invasive methods for measuring AS, in addition to cf-PWV, are arterial augmentation indices (AIx), the cardio–ankle vascular index, and the brachial–ankle pulse wave velocity, whose normal ranges are found in Table 1.

**Table 1:** Non-invasive methods for measuring AS and normal ranges (43).

AS measurement methods	Normal ranges
Cardio–ankle vascular index (CAVI)	8.01 ± 1.44
Brachial–ankle pulse wave velocity (ba-PWV)	12.93 ± 2.68 m/s
Carotid–femoral pulse wave velocity (cf-PWV)	6.53 ± 2.03 m/s
Augmentation index (AIx)	26.84 ± 12.79

## Clinical signs of vascular aging

Increased AS can be associated with advanced stages of BP and microvasculature pulsatility in organs such as the brain, heart, and kidneys, resulting in injury and functional deterioration of these systems. Therefore, premature VAg has become a health indicator and a predictor of target organ damage (2).

A progressive rise in AS has been found in disease conditions such as arterial hypertension (HTN), diabetes mellitus (DM), and dyslipidemia (17, 34, 44). In clinical practice, most HTN cases result from the aging of vascular wall cells (45). Increased BP is considered a clinical sign of aging (45) and, therefore, a determining factor in AS (18, 20). Studies have shown that, after age 50, SAP increases progressively while DAP decreases, widening the PP (28, 45), as a sign of aortic stiffness and VAg (45).

Type 2 DM is closely related to AS, as both cause significant functional and structural damage to the arterial wall. In type 2 DM, there is protein glycation, which causes vascular inflammation, increased oxidative stress, and, ultimately, endothelial dysfunction, resulting in AS (9, 17). CV disease has been shown to present two decades earlier in people with DM, which indicates that VAg manifests prematurely in this population (9, 46).

## Vascular aging phenotypes

In general, there are three vascular aging phenotypes, namely, normal vascular aging, early vascular aging (EVA), and supernormal vascular aging (SUPERNOVA).

### Normal vascular aging

There has been an interest in this natural phenomenon for almost a century (47). BP and vascular impedance determine blood flow. Vascular impedance depends on both the static and dynamic blood vessel pressure–volume ratio. Age is responsible (47, 48) for significant changes in the vascular structure and function, ultimately affecting the heart and other organs. Age is associated with large-vessel remodeling, characterized by a progressive increase in stiffness and changes in endothelial and VSMC function. Postmortem studies have shown that the aortic diameter in humans increases 15%–20% in people over the age of 65 (49). These changes are mainly found in elastic arteries and, therefore, have also been described in carotid, femoral, and even coronary arteries (50, 51). Stiffness is the hallmark of arterial age. Several studies have used non-invasive techniques to show that human arteries stiffen with age (52). Age is also associated with several endothelial function abnormalities. Cell rotation, DNA synthesis, and protein synthesis increase with age (53). Endothelial function is affected by age, with increased permeability to albumin, reduced macromolecular transport, and greater interaction between ECs and advanced glycation end products (54). Other endothelial function disorders occur with age, such as reduced release of vasodilating substances (PGI2, NO), as shown in an increased vascular tone. Furthermore, there are changes in other cells, such as the fibroblasts, which do not have an adequate cell replication rate, unlike the VSMCs, which can have greater proliferation due to a reduced expression of antiproliferative factors (e.g., TGF-β1) (55).

The effects of age on the vascular system should be differentiated from what occurs with atherosclerosis. Age is a physiological process that affects the entire vascular system, begins after sexual maturation, and leads to the enlargement of the arterial lumen. Over the last few decades, advances in vascular biology have provided a better understanding of this process.

### Early vascular aging

In 2008, the concept of EVA was first introduced (56). It refers to premature structural and functional alterations of the arterial wall. Its distinctive component is AS, which mimics the effects of aging and the inability of the wall to repair damage caused by the various mechanisms that induce stress (9). EVA can also provide information about the prior exposure of an individual to RFs (6). This phenotype is defined as a disjunction between chronological age and VA and an independent predictor of CV disease and death (17).

The cf-PWV must be measured to identify patients with EVA, as this is the hallmark of EVA (18); even so, there is no universal reference value because of differences between populations. Likewise, a person is considered properly diagnosed with EVA if the cf-PWV is higher than expected for their chronological age and sex (20). One study defined EVA as a VA more than 5.7 years greater than chronological age (20). The MARE study (57) defined it as a cf-PWV value above the age-interval-specific 90th percentile. Kotsis et al. (18) described it as a cf-PWV above the 95th percentile for sex and age.

The importance of this concept lies in its ability to identify individuals with high CVR early, without the need for classic RFs. The onset of this phenotype is related to genetic characteristics, environmental interactions, or exposure to various assaults, which alter and morphologically change the arterial tunica media (56). For an individual to have EVA, there must be an induction due to environmental and CV factors, genetic predisposition, and fetal programming (56). In addition to being elevated in individuals with premature coronary disease, another finding regarding cf-PWV is that it can also be elevated in first-degree relatives, showing the genetic nature of EVA (32).

### SUPERNOVA

The finding of an unusually low AS for chronological age and sex may be found in some individuals and is known as supernormal VAg or SUPERNOVA, referring to a large discrepancy between the chronological age and VA, in addition to being the phenotypic opposite of EVA (56). By definition, the AS values in these subjects are lower than those of the average population and healthy individuals (27). Although age-related increases in BP and AS are a part of the normal aging process, studies have identified a specific population in which age-related increases in BP and the development of atherosclerosis and AS are absent. At the same time, the SUPERNOVA concept may also be applied to individuals whose arterial elasticity has not been affected despite exposure to CV RFs. Therefore, no subclinical organ damage has been caused by this exposure. Thus, their risk of CV complications has not increased as much as in individuals without this phenotype (27, 56).

**Figure 3:**
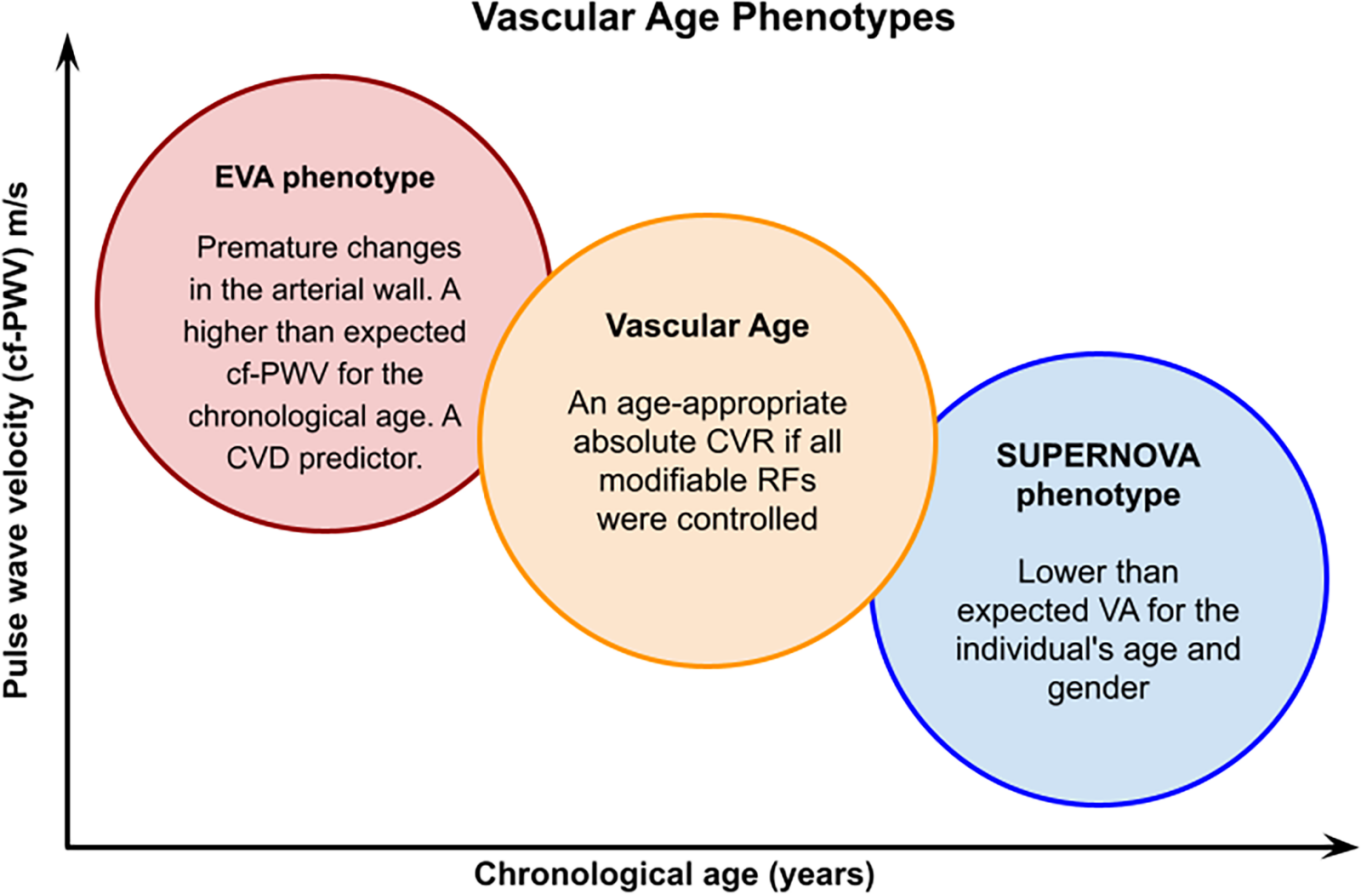
Vascular age phenotypes. A graphic representation of the vascular age phenotypes. In the EVA phenotype, individuals have elevated cf-PWV at an early age, while in the SUPERNOVA phenotype, older patients have a lower cf-PWV, despite having risk factors. EVA, early vascular aging; CVD, cardiovascular disease; CVR, cardiovascular risk; RFs, risk factors; AS, arterial stiffness.

According to VA, individuals with a SUPERNOVA phenotype have a 40% lower risk of CVD compared with individuals of the same chronological age but with normal VAg (9). According to a study by Bruno et al. (56) regarding SUPERNOVA, a significant difference between chronological age and VA has shown lower rates of CV events, regardless of CV RFs. Therefore, a SUPERNOVA is defined as a VA ≥6.2 years less than the chronological age. It is considered to be a protective factor against the effects of aging and CV RFs (Figure 3) (56).

Studying SUPERNOVA individuals is aimed at learning their genetic material to determine if a potential mechanism of action could be found to develop treatments aimed at prevention (56). The SUPERNOVA concept has profited translational research to find new mechanisms in controlling CVR and inspire the identification of key factors for reaching a healthy VAg, surpassing what can be achieved today with conventional RF control (9, 56).

### Vascular remodeling, AS, and inflammation

The widely accepted concept that phenotypic modulation or VSMC plasticity is the basis for many occlusive vascular diseases has recently been questioned. The finding of multipotent vascular stem cells, which repopulate the tunica media and may even form neointima after a vascular lesion, supports the hypothesis that multipotent vascular stem cell activation and differentiation, rather than mature VSMC undifferentiation, contribute to vascular remodeling and disease development (58). Furthermore, complex inter- and intracellular signaling pathways mediate vessel remodeling responses (59), which probably depend on the biomechanics of each individual cell. How the tensions and forces of individual cells and their intracellular structures and components are transmitted probably depends on the three-dimensional organization and interconnection of the ECM component and the cytoskeleton. Cells, proteins, multiple biochemical signals, and mechanical forces participate in the vascular remodeling process to maintain vascular integrity. The role of the ECM proteins, mainly the elastic fiber network, has been widely studied, and mechanical models have been proposed for CV development and the growth and remodeling of vessels in healthy adults (60). The VSMC integrin receptors, ROS, NADP with its five subunits (61), and ANG II or mineralocorticoid receptor activation play an important role in microvascular remodeling.

With advancing age, especially after the age of 30, the large arteries progressively lose their elastic properties (62). The medial arterial layer is made up of elastin, collagen, VSMCs, and a mucopolysaccharide matrix (63). These components are distributed in different proportions, going from central (elastic) to peripheral (muscular) arteries. With age, the large arteries are characterized by (1) a reduction in the elastin component and changes in elastin layer interactions, (2) an increase in the collagen and mucopolysaccharide matrix, (3) fewer VSMCs and increased stiffness, and (4) decreased proteolytic enzyme activity, which reduces the expected remodeling process of the vessel (64).

With normal arterial aging, the lumen diameter increases along with the thickness of the media in muscular arteries, with no significant change in the tunica media–lumen ratio (65). Increased brachial and central systolic pressure, as well as PP with aging, is due to the hardening of the large arteries, increasing the velocity of the reflected waves and producing greater geometric narrowing, causing more pressure wave reflection. In fact, normal aging exerts opposite effects on the proximal elastic arteries (which enlarge) and the distal muscular arteries (common femoral, brachial, and radial arteries) (which do not enlarge) (66).

**Figure 4:**
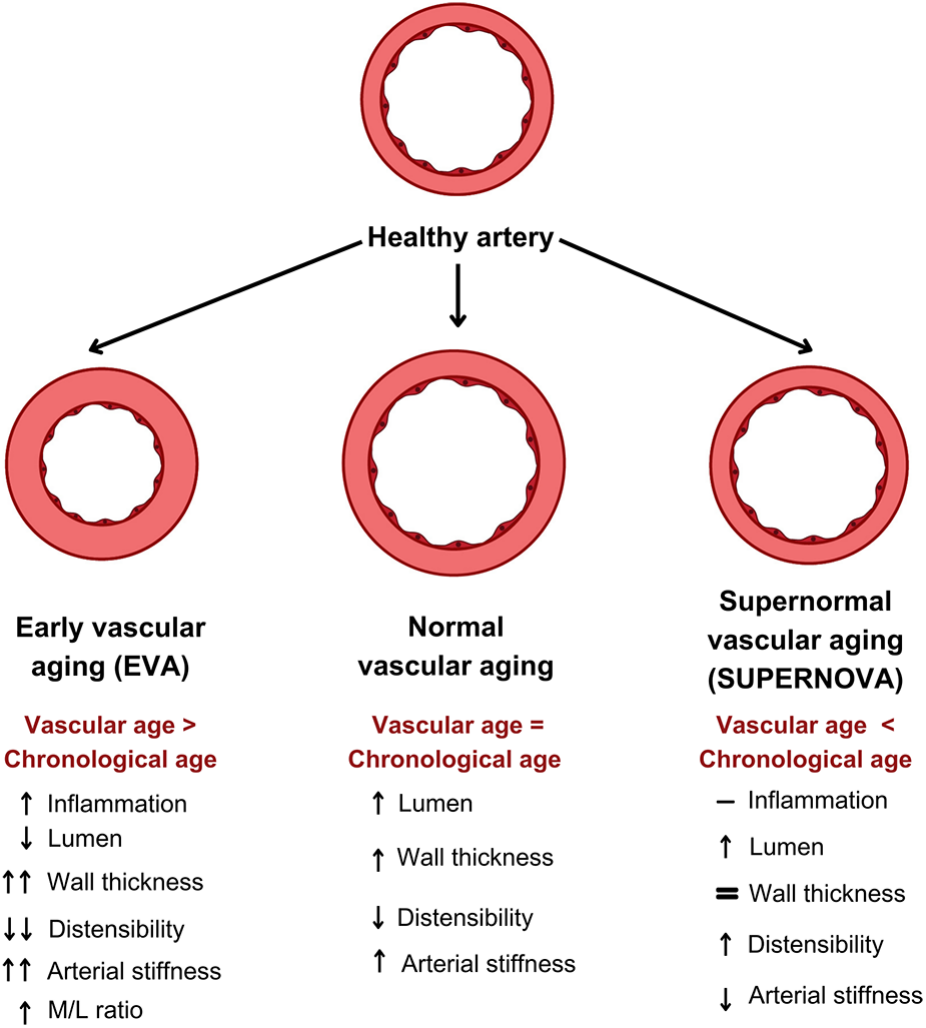
Arterial remodeling in the vascular aging phenotypes. A diagram of the different types of arterial remodeling with normal aging. The proximal large elastic arteries, such as the thoracic aorta, carotids, and abdominal aorta, enlarge (eccentric remodeling) and become stiffer with aging, while the distal medium-sized muscular arteries do not enlarge with age and their distensibility does not change. In both places, normal aging is associated with medial hypertrophy (hypertrophic remodeling). The EVA phenomenon is accompanied by a reduction in the lumen diameter and is associated with an increased media/lumen (M/L) ratio. With supernormal vascular aging (SUPERNOVA), the vascular area increases.

Chronic low-grade inflammation is well accepted as a significant determinant of large elastic artery and small muscular artery remodeling, especially in HTN (67, 68). This relationship between vascular inflammation and remodeling depends partly on the activation of the renin–angiotensin–aldosterone and endothelin systems. Chronic low-grade inflammation is also well accepted as a significant determinant in large artery remodeling. Large artery stiffness has been reported in various diseases associated with chronic low-grade inflammation, such as rheumatoid arthritis, systemic lupus erythematosus, systemic vasculitis, HIV, and inflammatory bowel disease. The EVA phenomenon could resemble what occurs with chronic inflammation or arterial HTN (Figure 4).

Wall remodeling in small arteries is mainly aimed at the ECM and triggered by increased oxidative stress and the production of growth factors such as TGF-β, PDGF, IGF, and bFGF (69). Adhesion molecules contribute to inflammation-induced remodeling of the small muscular arteries, especially by reorganizing the ECM–VSMC interactions and influencing the phenotypic modulation of VSMCs. Many cellular components, both from the innate and adaptive immune systems, mediate in this type of remodeling, namely, monocytes, macrophages, mastocytes, natural killer cells, and lymphocytes (70). Small artery remodeling induced by ANG II involves effector T cells such as T helper (Th) 1 (which produce IL-2, TNF, and interferon) and Th2 (which produce IL-4, IL-5, IL-6, and IL-10) lymphocytes, as well as Th17 lymphocytes and suppressor T cells. Chronic low-grade inflammation is also well accepted as a significant determinant of large artery remodeling. Large artery stiffness has been reported in various illnesses associated with chronic low-grade inflammation, such as EVA. Several mechanisms have been suggested, including endothelial dysfunction, ANG II activation of VSMC mineralocorticoid receptors, cellular release of various inducible MMPs, elastocalcinosis and accumulation of proteoglycans in the media, and, finally, adventitious immune cells and cytokines released by the vasa vasorum in response to vessel ischemia (71). Curiously, in patients with untreated essential HTN, aortic stiffness (evaluated through cf-PWV) was significantly correlated with high-sensitivity CRP and IL-6 (72).

### Vascular phenotypes and PWV

AS is a process related to aging and vascular, metabolic, cellular, and physiological deterioration. Over the last few years, it has been described as an independent predictor of CV mortality and CVD (73). It plays an important role in measuring the progression of various diseases such as DM, arterial HTN, CKD, retinopathies, and neuropathies.

That said, when the elastic arteries become stiff, the pressure gradient between the distal and proximal trees decreases. This increases transmission pressure, causing microcirculation damage and an increased risk of cerebrovascular disease and kidney damage (74, 75). The AS is measured using PWV and the aortic augmentation index (AIx).

Pulse wave velocity has been considered the gold standard for measuring AS. Although there is a continuous relationship between stiffness and events, the 2007 ESH/ESC Guidelines have suggested that PWV levels >12 m/s indicate target organ injury in hypertensive patients. A recent expert consensus even states that this value should be adjusted to a threshold of 10 m/s. Pulse wave velocity measurement holds a Class IIb, Level of Evidence B recommendation for patients with intermediate risk, as indicated in the latest 2013 ESH/ESC Hypertension Guidelines (42, 76). AS has shown an independent predictive value for CV events in patients with traditional FRs, allowing better identification of high-risk populations. It has been estimated that a 1.0 m/s increase in PWV is associated with a 14% increase in the risk of CV events (41).

Similarly, age-induced AS predominates in the proximal elastic arteries, with no effect on medium-sized distal arteries such as the brachial, radial, and femoral arteries (50). The structural alterations that occur with aging are associated with both active (reduced number of VSMC nuclei) and passive (reduced elastin-collagen ratio) changes in stiffness (77). In healthy middle-aged humans, PWV increases from 4–5 m/s in the ascending aorta to 5–6 m/s in the abdominal aorta and 8–9 m/s in the iliac and femoral arteries (78).

In fact, Segers et al. (79) showed a 15% increased PWV in healthy middle-aged subjects between 35 and 55 years old and an increased augmentation index, reflection ratio, and reflection magnitude, with no change in impedance.

Unlike optimal aging, which can be considered a balance between the harmful effects of mechanical, metabolic, and chemical tensions and the repair mechanisms, EVA is a defect in the repair mechanisms when faced with various tensions. EVA is typically found in young hypertensive patients with an elevated Young elastic modulus compared with older hypertensive patients or individuals with normal BP (80). Vascular aging and, more specifically, EVA are increasingly studied in humans using high-resolution non-invasive measurements of AS indices such as carotid intima-media thickness, central arterial pressure, and endothelial damage parameters. Specifically, normal and reference values for AS have been determined using cf-PWV measurement in 16,867 subjects and patients from 13 different centers in several European countries (42). Pulse wave velocity increases with age, and this age-related increase is more pronounced (that is, EVA) for higher BP categories and more CV RFs.

## Prevention and treatment

**Figure 5:**
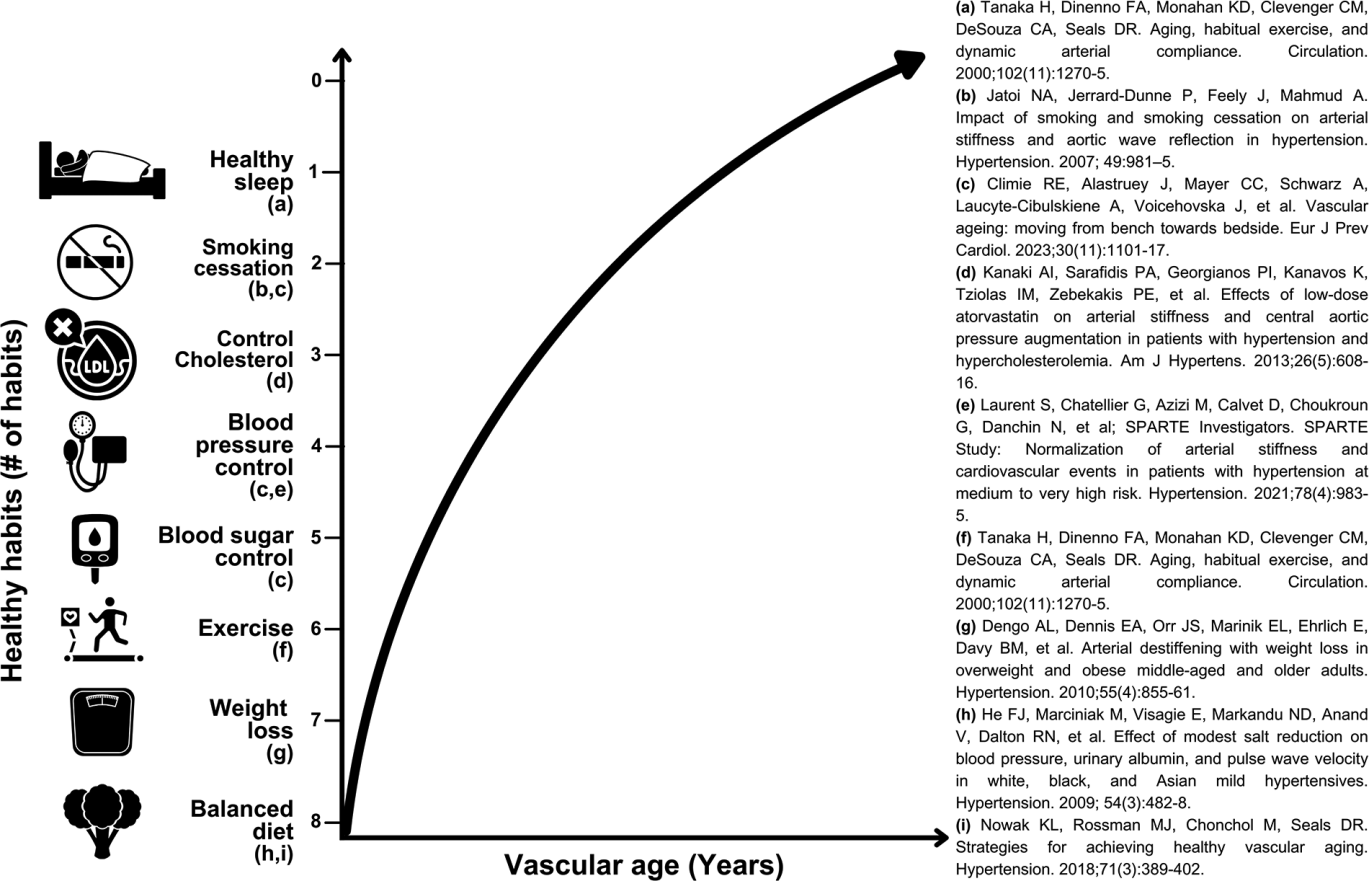
Theoretical correlation between the number of lifestyle habits and vascular age. A diagram of the theoretical relationship between the eight essential health habits recommended by the American Heart Association (81) and their impact on people's vascular age. The *X* axis shows the vascular age in years, and the *Y* axis shows the person's number of habits. A higher number of healthy habits are correlated with a lower vascular age. Icons adapted from Canva by authors licensed for use according to a Canva for Education license.

Conventional treatment is based on leading a healthy lifestyle and controlling RFs through evidence-based treatment for CVR reduction (44). Lifestyle changes continue to be the mainstay of interventions in people with EVA (32). Non-pharmacological therapies have been well described in many reviews and have proven to reduce AS. Among the most important of these are physical activity (especially aerobic exercise), smoking cessation, weight loss, and a healthy diet. In addition, less robust studies have suggested that low-sodium diets and a higher intake of powdered garlic, alpha-linolenic acid, fish oil, coffee, and green tea have proven beneficial in AS (32) (Figure 5).

Prior observational studies using beta blockers, calcium antagonists, and renin–angiotensin–aldosterone system inhibitor antihypertensive treatments have had a positive effect on vascular health (44), reducing AS and cf-PWV and exceeding the expected results in the BP itself (30, 44). Therefore, evaluating AS markers has been suggested for evaluating the effectiveness of antihypertensive treatment (17, 29). Other treatments are being studied, such as the pleiotropic, antioxidant, anti-inflammatory, vasodilating, and immunomodulating effects of statins (25, 44). Finally, metformin influences the mechanisms affected by biological aging by activating AMPK and inhibiting the mTOR pathway, reducing endothelial dysfunction and AS (4, 82, 83).

In addition, renin–angiotensin–aldosterone system inhibitors are proven to increase the amounts of nicotinamide phosphoribosyltransferase and sirtuin 3, playing a role in controlling vascular aging (5, 84). Metformin also exerts an epigenetic action, increasing sirtuin 1 levels, which is one of the molecular pathways proposed by Kreutzenberg et al. to explain increased cellular longevity in patients taking this medication (85). New therapies have been developed, such as spermidine, which prevents hyperacetylation of histone H3, and resveratrol, which acts as a sirtuin 1 agonist, slowing the progression of vascular aging (35, 86).

Classic risk score calculators such as Framingham or SCORE can be found to predict CVR and CV events in individuals with traditional CV RFs such as age, cholesterol levels, SAP, DM, and smoking (6, 10). Although the effectiveness of these calculators has been proven, they may fail to predict CV events in non-traditional risk groups (6), such as individuals with EVA. As a result, while these are useful tools for clinical practice, it is very important to manage each patient individually.

## Conclusion

VA is an innovative concept in the study of CVR, which has proven to have great potential for developing new treatments aimed at CVD prevention. AS is the main finding in VAg, causing increased BP and greater pulsatility in the microvasculature. Two VA phenotypes have been reported, namely, EVA, in which individuals have premature molecular and structural changes in the arterial wall, and SUPERNOVA, in which there is an unusually low AS for the chronological age. Therefore, SUPERNOVA is a condition that allows us to research treatment alternatives that can delay AS and, in turn, reduce CVR.

VA helps identify individuals with the risk of developing CVD who would go unnoticed using other methods or stratification scales. This is a promising field in the identification of CV RFs which requires further studies to evaluate AS in various populations and the phenotypic prevalence of VAg.

## Footnotes

^1^Windkessel effect: a process by which elastic arteries, mainly the aorta, store up to 50% of the ejection volume during systole, and when the arterial pressure drops during diastole, the coronaries maintain a continuous and uninterrupted blood flow (19).
